# Compartmentalized citrullination in Muller glial endfeet during retinal degeneration

**DOI:** 10.1073/pnas.2121875119

**Published:** 2022-02-23

**Authors:** Sarah I. Palko, Nicholas J. Saba, Elias Mullane, Benjamin D. Nicholas, Yosuke Nagasaka, Jayakrishna Ambati, Bradley D. Gelfand, Akihito Ishigami, Paola Bargagna-Mohan, Royce Mohan

**Affiliations:** ^a^Department of Neuroscience, University of Connecticut Health Center, Farmington, CT 06030;; ^b^Department of Ophthalmology, University of Virginia Health System, Charlottesville, VA 22908;; ^c^Center for Advanced Vision Science, University of Virginia Health System, Charlottesville, VA 22901;; ^d^Department of Biomedical Engineering, University of Virginia, Charlottesville, VA 22908;; ^e^Molecular Regulation of Aging, Tokyo Metropolitan Institute of Gerontology, Tokyo 173-0015, Japan

**Keywords:** Muller glial endfeet, citrullination, retinal degeneration, gliosis, GFAP

## Abstract

Muller glia (MG) play a central role in reactive gliosis, a stress response associated with rare and common retinal degenerative diseases, including age-related macular degeneration (AMD). The posttranslational modification citrullination​ targeting glial fibrillary acidic protein (GFAP) in MG was initially discovered in a panocular chemical injury model. Here, we report in the paradigms of retinal laser injury, a genetic model of spontaneous retinal degeneration (JR5558 mice) and human wet-AMD tissues that MG citrullination is broadly conserved. After laser injury, GFAP polymers that accumulate in reactive MG are citrullinated in MG endfeet and glial cell processes. The enzyme responsible for citrullination, peptidyl arginine deiminase-4 (PAD4), localizes to endfeet and associates with GFAP polymers. Glial cell–specific PAD4 deficiency attenuates retinal hypercitrullination in injured retinas, indicating PAD4 requirement for MG citrullination. In retinas of 1-mo-old JR5558 mice, hypercitrullinated GFAP and PAD4 accumulate in MG endfeet/cell processes in a lesion-specific manner. Finally, we show that human donor maculae from patients with wet-AMD also feature the canonical endfeet localization of hypercitrullinated GFAP. Thus, we propose that endfeet are a “citrullination bunker” that initiates and sustains citrullination in retinal degeneration.

Muller glia (MG) are the principal reactive cells of the retina that respond to stress via a process known as reactive gliosis (1). The posttranslational modification (PTM) citrullination (2) of glial fibrillary acidic protein (GFAP) can occur in reactive MG, where citrullination driven by peptidyl arginine deiminase-4 (PAD4) expression is closely regulated early during gliosis in a mouse model of corneal alkali injury (3, 4). The conversion of arginine residues on GFAP could promote its depolymerization (5), or potentially, citrullinated GFAP could also be cleaved (6), and citrullinated fragments may serve as autoimmunogens (7). Given the blunt nature of alkali injury, whether MG elicit hypercitrullination in other injuries or retinal diseases is unknown. Therefore, we used three models of retinal pathology to investigate the PAD4-citrullination axis in MG.

## Results and Discussion

**Fig. 1.**
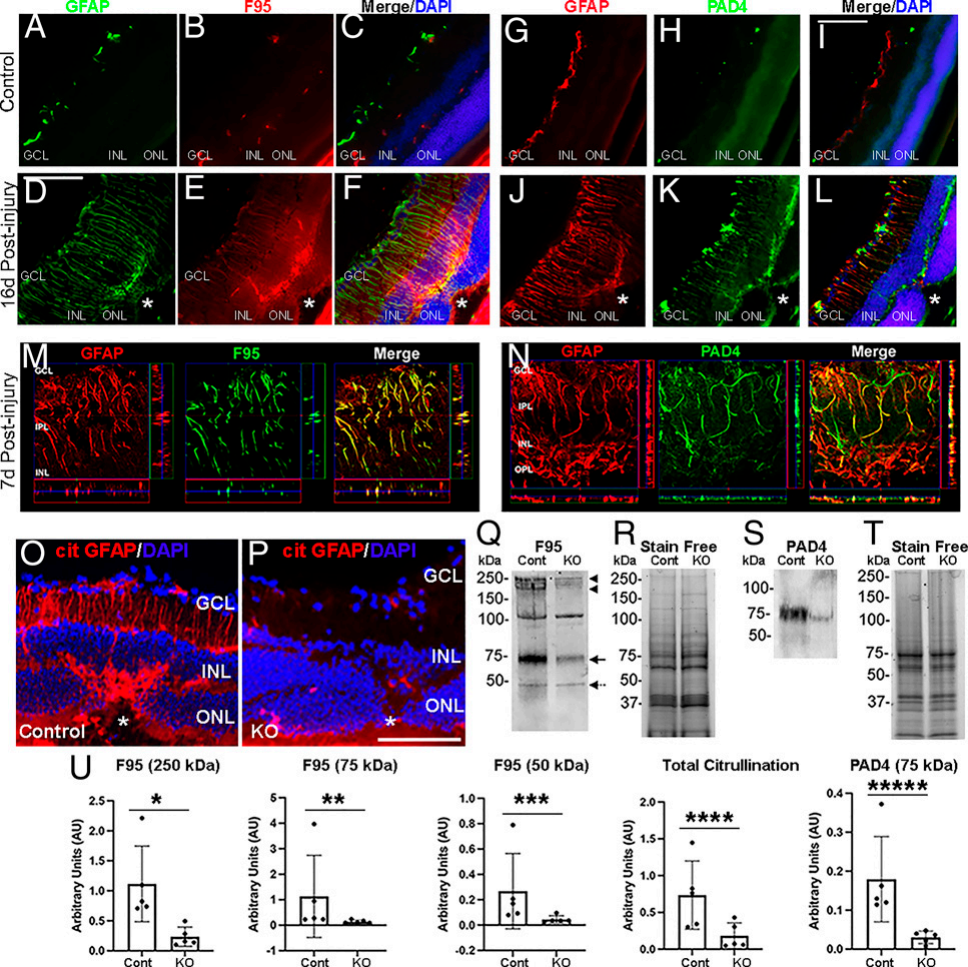
Citrullination and PAD4 in reactive MG after laser injury. (*A–F*) Representative images of retinal sections from uninjured and postinjured mice immunostained for GFAP (green) and citrullination (F95, red). Nuclei were visualized with DAPI (blue). Asterisks demark laser injury site. (*M*) Orthogonal projections of confocal *z-*stacks stained for GFAP (red) and F95 (green) at high magnification. (*G–L*) Representative images of retinal sections from uninjured and postinjured mice immunostained for GFAP (red) and PAD4 (green). (*N*) Orthogonal projections of confocal *z-*stacks stained for GFAP (red) and PAD4 (green). (Scale bars, 105 μm.) (*O* and *P*) Representative images of retinas from laser injured control and PAD4cKO (KO) mice immunostained for cit-GFAP. GCL, ganglion cell layer; INL, inner nuclear layer; ONL, outer nuclear layer. WB analysis of retinal extracts subjected to denaturing and reducing conditions from laser-injured control and KO retinas probed for citrullination (F95; *Q*) and PAD4 (*S*). (*U*) Quantitation of F95 reactive bands and PAD4 were normalized to stain-free gels (*R* and *T*), respectively (*n* = 5 blots; error bars are SD of mean; *P = 0.004, **P = 0.0159, ***P = 0.0159, ****P = 0.0278, *****P = 0.004) using one-way ANOVA.

We first employed the laser-induced mouse model of accelerated choroidal neovascularization (in short, laser injury), a pathological process that occurs in wet-AMD and pathologic myopia, to assess retinal gliosis (8). We detected hypercitrullination of glial filaments in reactive MG at the endfeet and along cell processes reaching the outer nuclear layer (Fig. 1 *A–F*, and *M*). PAD4 shows a similar pattern of compartmentalized staining at the endfeet and along glial filaments (Fig. 1 *G–L*, and *N*). To test whether PAD4 is responsible for MG hypercitrullination, we selectively ablated *Padi4* gene expression by crossing *GFAP-Cre^ERT2^* with *Padi4^flox/flox^* mice and administered tamoxifen to induce PAD4 deficiency in glial cells (PAD4 conditional knockout mice [PAD4cKO]) (*SI Appendix*). Immunostaining of retinas from control and PAD4cKO mice subjected to laser injury (*SI Appendix*) revealed loss of citrullinated GFAP (cit-GFAP) in PAD4cKO retinas compared to control injured retinas as detected by a site-specific (R270 and R416) anticitrullinated GFAP antibody (4) (Fig. 1 *O* and *P*). Western blot (WB) analysis (Fig. 1*Q*) confirmed that PAD4cKO retinas showed significant fourfold reduction of the total hypercitrullinated species (Fig. 1*U*), including that of protein species around 250 kDa (Fig. 1*Q*, arrow heads), 75 kDa (Fig. 1*Q*, arrow), and 50 kDa GFAP (Fig. 1*Q*, dotted arrow), but not the ∼100-kDa band. This attenuation paralleled the near sixfold reduction of PAD4 protein levels in PAD4cKO retinas compared to injured controls (Fig. 1*S*).

**Fig. 2.**
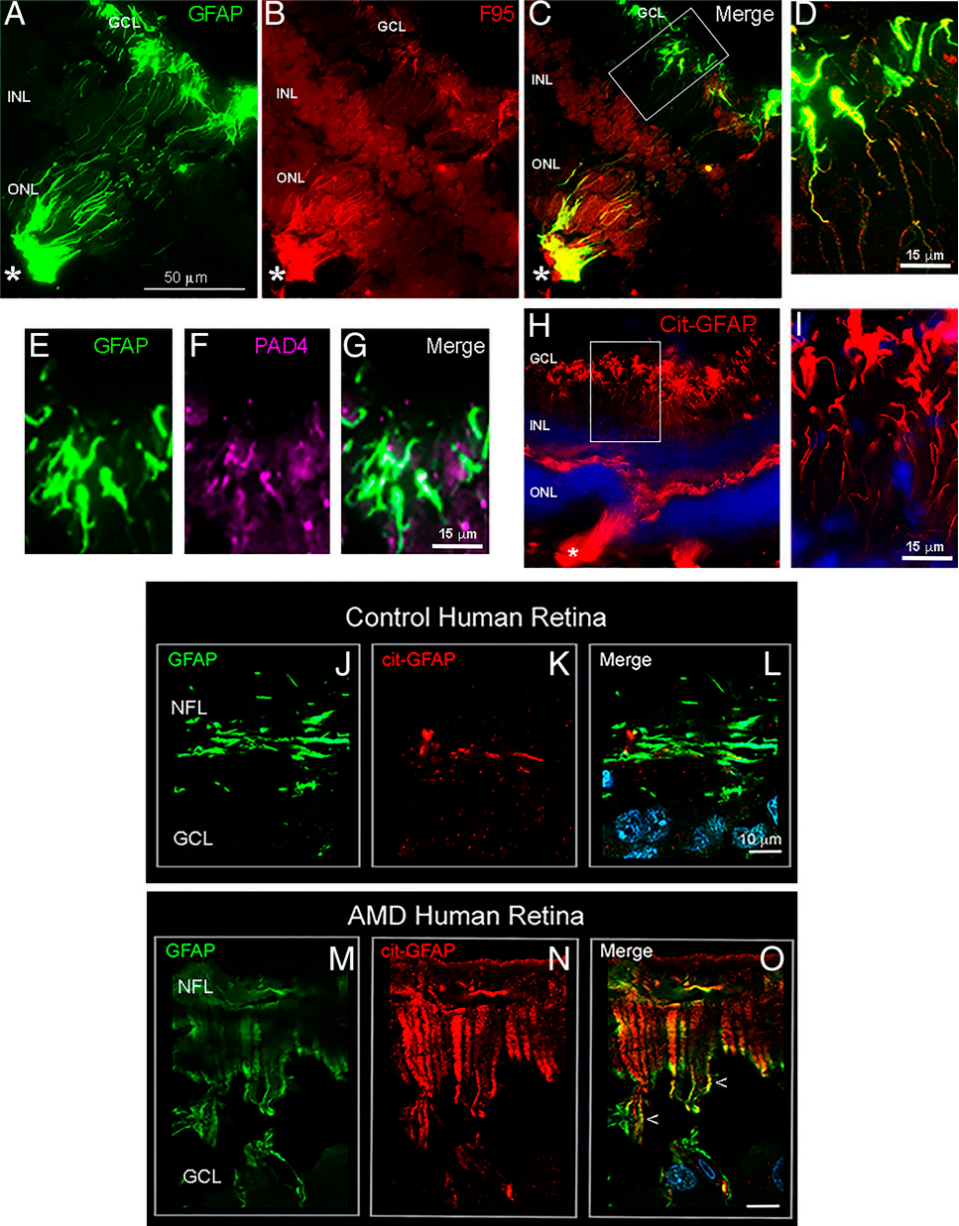
Citrullinated GFAP in MG endfeet of JR5558 mouse and human wet-AMD diseased retinase. Localization of cit-GFAP in JR5558 mouse retinas and human wet-AMD macula. One-month-old JR5558 mouse retinas were immunostained for GFAP, citrullination (F95), cit-GFAP, and PAD4. Lesion-specific staining for GFAP (*A*; green) overlaps with staining with F95 (*B*; red) and is seen in the merged image (*C*). Representative higher magnification images show F95 colocalization with GFAP in endfeet (*D*). Representative higher magnification images of GFAP staining (*E*; green) and PAD4 (*F*; magenta) showing colocalization (*G*). Cit-GFAP staining in a 2-mo-old JR5558 mouse retina (*H*) with higher magnification revealed at endfeet (*I*). Asterisks mark the site of spontaneous retinal lesions. (*J*–*O*) Representative tissue sections from 89-y-old wet-AMD and age-matched control donor maculae stained for GFAP (green) and cit-GFAP (red). The endfeet expression of cit-GFAP in wet-AMD macula is revealed in high magnification images showing overlap with GFAP staining (*M–O*; arrowheads), whereas staining in controls is low (*K*) and overlaps minimally with GFAP (*L*). NFL, nerve fiber layer; GCL, ganglion cell layer; INL, inner nuclear layer; ONL, outer nuclear layer. (Scale bar, 10 μm.).

Next, we investigated citrullination in a genetic model of spontaneous outer retinal degeneration. The *Crb1^rd8^*/*Jak3 ^m1J^* double mutant mouse (JR5558 line) develops retinal lesions leading to adult-onset retinal degeneration (9). Retinal lesions in 1-mo-old JR5558 mice show lesion-specific GFAP overexpression with abundant staining of both hypercitrullinated species (Fig. 2*A–D*) and cit-GFAP marking the entire length of the MG cell processes (Fig. 2 *H* and *I*). PAD4 also localizes to the MG endfeet region overlapping GFAP (Fig. 2 *E–G*), further exemplifying that the PAD4–citrullination axis is also orchestrated during gliosis in a model of rare retinal disease.

Finally, we analyzed retinal tissue from advanced human wet-AMD donor eyes for citrullination (10) (*SI Appendix*). The control age-matched donor samples revealed the expected GFAP staining in the ganglion cell layer astrocytes, with some filamentous GFAP staining suggestive of minimal age-related gliosis (Fig. 2*J*). The wet-AMD retinas revealed considerable retinal swelling and increased staining for GFAP that featured localization to MG endfeet and through the cell processes (Fig. 2*M*) (11). Staining for cit-GFAP revealed low levels in the astrocyte layer of controls (Fig. 2*K*), but in wet-AMD maculae the striking abundance of cit-GFAP was particularly noticeable in the endfeet and along cell processes, overlapping with GFAP staining (Fig. 2*N*). Taken together, our findings from mouse models and human retinal degenerative disease reveal an association of endfeet citrullination with pathology. Rigorous validation of this potential human disease–related biomarker should be established using larger sample numbers.

A parsimonious view of citrullination coordination with gliosis builds on the idea that *Gfap* mRNA transport to the endfeet for its localized translation (12) and assembly into polymeric forms would allow for PAD4 to become associated during GFAP polymerization. This suggests that the enzyme and substrate production may be coregulated. One may postulate that PAD4 is synthesized within endfeet and further activated in situ by the influx of high calcium concentrations via the endoplasmic reticulum calcium sensor (13). As such, this spatially defined, stress-induced PTM could occur concurrently with phototransduction, where such an arrangement of compartmentalized stress response would afford visual processing even during the course of an injury/stress response.

In summary, compartmentalized mRNA translation and PTMs are becoming more broadly recognized in biology, especially in highly polarized neurons and astrocytes (14). We propose the term “citrullination bunker” for the MG endfeet, because this PTM becomes engaged as a polarized response following an insult and continues into chronic pathological stages. Thus, [hyper]citrullination could serve to modulate retinal gliosis through depolymerization of citrullinated GFAP filaments (5) or to transport activated PAD4 to other cellular substrates for citrullination (e.g., nuclear histones) (15). Finally, chronic hypercitrullination may eventually alter retinal immunity through production of anticitrullinated peptide antibodies, as in rheumatoid arthritis (7).

## Materials and Methods

See *SI Appendix*, *Extended Methods* for more details.

### Mice and Laser Injury.

C57BL/6J mice and PAD4cKO mice were subjected to laser injuries as described (16) (see also *SI Appendix*). JR5558 mice were purchased from The Jackson Laboratory.

### Western Blot and Immunofluorescence Staining Analyses.

Mouse retinas from control and injured mice were subjected to WB and immunostaining (*SI Appendix*). Human donor eyes from deidentified subjects (*n* = 4 wet-AMD; *n* = 3 normal), procured within 8 h of death, were dissected in buffered formalin and then the posterior eye cup was validated for disease stage (*SI Appendix*). Circular 8-mm tissue punches of the macula were cryopreserved and sectioned. Six different sets of sections from each donor eye were obtained and randomly chosen for analysis to ensure adequate representation of the macula tissues.

### Statistical Analysis.

Gel bands for WBs were normalized against stain-free gels using FIJI software. Data were analyzed by one-way ANOVA for significance of *P* < 0.05 using PRISM software.

## Supplementary Material

Supplementary File

## Data Availability

All study data are included in the article and/or *SI Appendix*.
