# Supplementary material for: Compartmentalized citrullination in Muller glial endfeet during retinal degeneration
Source: Proc Natl Acad Sci U S A. 2022 Feb 23;119(9):e2121875119. doi: 10.1073/pnas.2121875119 (PMC8917347; doi:10.1073/pnas.2121875119)
Supplement: Supplementary File [file pnas.2121875119.sapp.pdf]

PNAS
www.pnas.org

Supplementary Information for

Compartmentalized Citrullination in Muller Glial Endfeet during Retinal Degeneration

Sarah I. Palko[a], Nicholas J. Saba[a], Elias Mullane[a], Benjamin D. Nicholas[b], Yosuke Nagasaka[b,c], Jayakrishna Ambati[b,c], Bradley D. Gelfand[b,c,d], Akihito Ishigami[e], Paola Bargagna-Mohan[a], and Royce Mohan[a, 1]

[a]Neuroscience, University of Connecticut Health Center, Farmington, CT 06030; [b]Department of Ophthalmology, University of Virginia Health System, Charlottesville, VA 22908; [c]Center for Advanced Vision Science, University of Virginia Health System, Charlottesville, VA 22901; [d]Department of Biomedical Engineering, University of Virginia, Charlottesville, VA 22908; [e]Molecular Regulation of Aging, Tokyo Metropolitan Institute of Gerontology, Tokyo 173-0015, Japan.

[1]Corresponding author: Royce Mohan
Email: mohan@uchc.edu

This PDF file includes:

Supplementary text
SI References

Supplementary Text

Extended Methods

Generation of PAD4cKO mice
B6.Cg-Tg(GFAP-cre/ERT2)505Fmv/J (Stock No: 012849; Jax labs, Maine; termed *GFAP-Cre*^{ERT2} in short) male and female mice were obtained and breeding colonies established. B6.Cg-*Padi4*$^{tm1.2Kmow}$/ (Stock No: 026708; Jax Labs, Maine; termed *Padi4*$^{flox/flox}$ in short) male and female mice were obtained and breeding colonies established. Next, the *GFAP-Cre*^{ERT2} line was crossed with *Padi4*$^{flox/flox}$ line to achieve the desired double transgenic PAD4cKO mouse line*.* Mice were genotyped by polymerase chain reaction to establish their genetic identities.

JR5558 mice
B6.Cg-*Crb1*^{rd8} *Jak3*^{m1J}/Boc (Stock No: 005558; Jax labs; also called JR5558) mice were obtained (2 females, 1 male) and retinas imaged (see also below) at 1 month and 2 months of age using the Micron III Imaging System (Phoenix, AZ). This allowed us to visualize the spontaneous development of multiple retinal lesions prior to euthanasia and subsequent analysis by immunofluorescence staining.

Mice and Laser Injury
Mice were provided anesthesia with an intraperitoneal (IP) injection of ketamine/xylazine and placed on a warming blanket. Phenylephrine hydrochloride and tropicamide were used together to dilate the pupil. An application of GenTeal Tears lubricant eye gel (Alcon Laboratories, Inc) was immediately applied to the cornea to prevent tissue drying and additionally applied prior to imaging serving as a contact lens for the imaging objective of the Micron III Imaging System (Phoenix, AZ). Once the retinal pigment epithelium (RPE) was brought into focus, the red-light guide laser was employed to focus the beam on the retinal location for delivery of the laser burn (Meridian Merilas Nd-Yag 532 alpha green laser photocoagulator) integrated with the Micron III imaging system. The laser beam of 50 μm in diameter was employed to deliver 250 mW at 100 msec focal lesions. Six such laser lesions were delivered to the RPE in an equally spaced pattern surrounding the optic nerve. Care was taken to position the laser burns avoiding retinal blood vessels. Mice were subsequently recovered on warming blankets until fully ambulatory and returned to their housing units. At different time points, injured and control mice were euthanized by CO_2 inhalation and cervical dislocation. Immediately after sacrifice, eyes were enucleated, frozen on dry ice, and maintained at -80°C.

Western Blot (WB) Analysis
Cornea, lens and vitreous were carefully separated from the posterior eye cups of mouse eyes. Protein from the retina/choroid tissue pooled from 2 eyes/sample was extracted in RIPA buffer (25 mM Tris-HCl, pH 7.6, 150 mM NaCl, 1% NP-40, 1% sodium deoxycholate, 0.1% Sodium dodecyl sulfate) containing a cocktail of protease inhibitors (Roche, Indianapolis, IN) plus 1 mM sodium vanadate and 1 mM phenylmethyl sulfonyl fluoride. Samples were incubated on ice for 25 minutes with occasional vortexing and then centrifuged at 14,000 x *g* for 5 minutes at 4°C. The supernatant was removed, sheared through the tip of a p200 pipette, and centrifuged again at 14,000 x *g* for 5 minutes. The supernatants were normalized after adjusting to protein content (Bradford assay). Protein samples were denatured by boiling at 100°C for 10 min in presence of freshly added reducing agent ß-mercaptoethanol into Laemmeli buffer prior to loading on precast Criterion TGX-Stain-Free gels (BIO-RAD, CA). Gels were imaged (ChemiDoc XRS+ Gel Imaging System, BIO-RAD, CA) for protein loading prior to transfer to membranes using a semi-dry apparatus (Trans-Blot Turbo Transfer System, BIO-RAD, CA). Blots were blocked in 5% non-fat dry milk in Tris-buffered saline for 1-2 h at 4°C, followed by incubation overnight at 4°C in antibody solution prepared in blocking buffer.

Immunofluorescence (IF) staining

For murine samples, eyes were enucleated immediately after euthanasia, rinsed in phosphate buffered saline (1X PBS), and embedded in optimum cutting temperature compound (OCT; Tissue-Tek, Radnor, PA), and stored at -80°C. Briefly, 12 micron cryosections were air dried for 30 minutes at room temperature, fixed with 4% paraformaldehyde/PBS for 10 minutes, and then washed with PBS three times for 5 minutes each. Samples were probed with primary antibodies diluted in DAKO background reducing solution (Agilent Tech, Santa Clara, CA, Cat# S3022). After three washes with PBS slides were then incubated with secondary antibodies for 1 hour at room temperature (RT), and washed 3 times for 10 minutes before imaging.

For human samples, deidentified human donor eyes (n=3 controls and n=4 wet-AMD; range 77 to 89 years of age), procured within 8 hours of death, were dissected in buffered formalin and then the posterior eye cup imaged using Spectral Domain-Optical Coherence Tomography (Spectralis; Heidelberg Engineering) as described (Pang et al., Ophthalmology 2015). 8.2 mm, 7 line scans on macular crossing the optic disc were obtained. 30° x 30° autofluorescence fundus images were obtained with 488 nm argon blue laser and averaged from 9 images. 8 mm tissue punches of the macula were collected and cryopreserved for immunostaining. 12 micron sections of cryopreserved human maculae were collected on slides and dried overnight at 37°C. Sections were fixed in 4% PFA/PBS for 15 minutes, stained with DAPI for five minutes, and then blocked 3 hours at 37°C. Slides were incubated with primary antibodies at 4°C and then secondary antibodies for 1 hour at room temperature. Sections were imaged using confocal microscopy. Additional slides were dried overnight and fixed in 4% PFA/PBS for 15 minutes and stained with DAPI before being imaged by confocal microscopy for assessment of tissue autofluorescence. The imaging parameters employed for detection of antibodies were maintained for autofluorescence. Six different sets of sections from each donor eye were obtained and randomly chosen for analysis to ensure adequate representation of the macula tissues.

Antibodies used and conditions

1. Anti-GFAP, Abcam Cat# ab4674, Polyclonal IgG Chicken; IF: 1:500, (3 h @ 37°C).
2. Anti-PAD4, Abcam Cat# ab50247, Polyclonal IgG Rabbit, IF: 1:100 (3h @ 37°C)
3. Anti-PAD4, Biolegend Cat# 684202, Monoclonal IgG Mouse, WB: 1:1000
4. Anti-F95, Millipore Cat# MABN328, Monoclonal IgM Mouse, IF 1:200 (O/N @ 4°C); WB: 1:100
5. Anti-cit-GFAP, CTGF-1221 (Ishigami Lab), Monoclonal IgG Mouse, IF: 1:100, (3hr @37°C)
6. Anti-Rabbit HRP Conjugated, Santa Cruz Cat# Sc-2301, IgG Goat, WB: 1:1000
7. Anti-Mouse HRP Conjugated, Santa Cruz Cat# Sc-2302, IgG Goat, WB 1:1000
8. Anti-Mouse HRP Conjugated, Jackson Immunoresearch Cat# 115–035–075, IgM Goat, WB 1:1000
9. Anti-Mouse, Alexa 594, Thermofisher Cat# 11032, IgG Goat, IF: 1:500 (1h @ RT)
10. Anti-Chicken, Alexa 488, Thermofisher Cat# 32931, IgY Goat, IF: 1:500 (1h @ RT)
11. Anti-Rabbit, Alexa 647, Thermofisher Cat# 21245, IgG Goat, IF: 1:500 (1h @ RT)
12. Anti-Mouse, Alexa 594, Thermofisher Cat# 21044, IgM Goat, IF: 1:500 (1h @ RT)

Confocal microscopy

Stained sections were examined and captured on a Ziess LSM800 confocal microscope. Mouse tissue was imaged and orthogonal projections were generated to analyze protein colocalization. Human tissue was imaged by confocal microscopy and maximum intensity projection was created from Z-stacks.

Rigor and Reproducibility

To randomize the analysis, different batches of C57Bl/6 mice were procured and subjected to experimental injury over a 2.5-year-period. To control for technical variability in the experimental injury model, three trained individuals performed the laser injuries at different times in this study, and as such, eyes from such separate experiments were collectively used for analysis. Also, different batches of PAD4cKO mice and littermates were derived and subjected to experimental

injury. Mice of both sexes were employed, and the sex of mice were recorded, although the analyses was performed masked to the sex of the animal. As no significant differences were noted in the results after unmasking, these findings represent responses of male and female mice to the laser injury.

SI References

1. C. E. Pang, J. D. Messinger, E. C. Zanzottera, K. B. Freund, C. A. Curcio, The Onion Sign in Neovascular Age-Related Macular Degeneration Represents Cholesterol Crystals. *Ophthalmology* **122**, 2316-2326 (2015).
